# Broadband and Ultra-Low Threshold Optical Bistability in Guided-Mode Resonance Grating Nanostructures of Quasi-Bound States in the Continuum

**DOI:** 10.3390/nano11112843

**Published:** 2021-10-26

**Authors:** Xin Li, Zhongshuai Zhang, Yanyan Huo, Lina Zhao, Qingyang Yue, Shouzhen Jiang, Huawei Liang, Yuanmei Gao, Tingyin Ning

**Affiliations:** 1 Shandong Provincial Engineering and Technical Center of Light Manipulations, Shandong Provincial Key Laboratory of Optics and Photonic Device, School of Physics and Electronics, Shandong Normal University, Jinan 250358, China; 2019020515@stu.sdnu.edu.cn (X.L.); 2020020556@stu.sdnu.edu.cn (Z.Z.); lnzhao@sdnu.edu.cn (L.Z.); qingyangyue@sdnu.edu.cn (Q.Y.); jiang_sz@126.com (S.J.); 2 Shenzhen Key Laboratory of Laser Engineering, College of Physics and Optoelectronic Engineering, Shenzhen University, Shenzhen 518060, China; hwliang@szu.edu.cn

**Keywords:** bound states in the continuum, optical bistability, guide-mode resonance grating

## Abstract

We model optical bistability in all-dielectric guide-mode resonance grating (GMR) nanostructures working at quasi-bound states in the continuum (BICs). The complementary metal-oxide-semiconductor (CMOS) compatible material silicon nitride (SiN) is used for the design of nanostructures and simulations. The ultra-low threshold of input intensity in the feasible nanostructure for nanofabrication is obtained at the level of ~100 W/cm^2^ driven by quasi-BICs. Additionally, the resonance wavelength in the GMR nanostructure can be widely tuned by incident angles with the slightly changed *Q*-factor that enables the optical bistable devices to work efficiently over a wide spectrum. The impact of the defects of grating that may be introduced in the fabrication process on the optical properties is discussed, and the tolerance of the defects to the optical performance of the device is confirmed. The results indicate that the GMR nanostructures of broadband and ultra-low threshold optical bistability driven by quasi-BICs are promising in the application of all-optical devices.

## 1. Introduction

Optical bistability is a fundamental optical phenomenon that has potential applications in all-optical switching, transistors, logic gates, etc. [1]. The nonlinear optical materials with the intensity dependent refraction or absorption are essential to realize the bistable behavior. However, the optical nonlinearity in the materials is commonly very weak, and the resonance cavity as a feedback system is conventionally required to enhance the local field and thus, reduce the power consumption [1]. Traditionally, the photonic crystal nanocavity [2,3], Fabry–Pérot cavity [4,5], ring resonator [6,7], and surface plasmon resonance system [8,9,10,11] are employed for the cavities. The high *Q*-factor and small mode volume *V* of the resonance cavities are responsible for the operation of optical bistability at a low power.

The all-dielectric guided-mode resonance (GMR) grating nanostructure is one of the most important nanoresonators for efficient nonlinear photonic devices [12,13,14,15,16,17]. The GMR nanostructure fabricated in Kerr nonlinear materials was studied for optical bistable devices [18]. The field in the waveguide layer can be dramatically enhanced at the resonance wavelength to realize low-power optical bistability. Further, the GMR has high degree of optical tunability for devices, such as wavelength, polarization, phase and intensity [18]. Most importantly, GMR nanostructures of large-scale patterning can be fabricated by laser interference lithography or electron-beam lithography combining with nanoimprint lithography for high-throughput and cost-efficient requirements [18]. The theoretical study on optical bistability in corrugated waveguides dates back more than 30 years [19,20]. Numerous works were conducted to increase the *Q* factor to lower the intensity threshold of optical bistability in GMR, such as the coupled GMR nanostructures of air gaps [21], GMR of double-layer gratings [22], and low-index material embedded in the waveguide layer [23]. However, these methods are somewhat complicated for nanofabrication, and the intensity threshold of optical bistability is still on the high-level of MW/cm^2^. In recent years, the optical BICs of ultra-high *Q*-factor has drawn much attention, due to the extremely confined light field to dramatically enhance the light–matter interaction for sensing, nano-lasering and nonlinear optics [24]. The optical multistable behavior in a one-dimensional Si photonic crystal slab of nonlinear BICs was predicted at the pump power of several μW/cm^2^ [25]. However, the effective refractive index modulation amplitude *δn* is used in the article, not the exact distribution of refractive index modulation related to local intensity, which can sensitively affect the optical transmission and reflection. The evolution of reflection or transmission with the change in input intensity is also lacking. So, further works are needed to study the optical bistable behavior in the resonance structures of BICs.

Recently, the GMR nanostructures consisting of a four-part grating layer of quasi-BICs with ultra-high *Q* factors were investigated for Goos–Hänchen shift and harmonic generation [26,27]. In this paper, we further investigate the optical bistability in GMR nanostructures of quasi-BICs. The complementary metal–oxide–semiconductor (CMOS) compatible material silicon nitride (SiN) is employed to design the GMR structure. The temporal coupled-mode theory (TCMT) and finite element method (FEM) are employed to study the optical behaviors in nonlinear GMR nanostructures. Aided by the giant enhanced local field in the waveguide at the quasi-BICs, the optical bistability of ultra-low threshold intensity at the level of ~100 W/cm^2^ is predicted. Importantly, the optical bistable devices working in a broad band can be realized by changing the incident angles to tune the resonance wavelength of the nanostructures. The effects of typical defects of grating on the performance of the optical devices are discussed.

## 2. Numerical Model and Material Parameters

**Figure 1:**
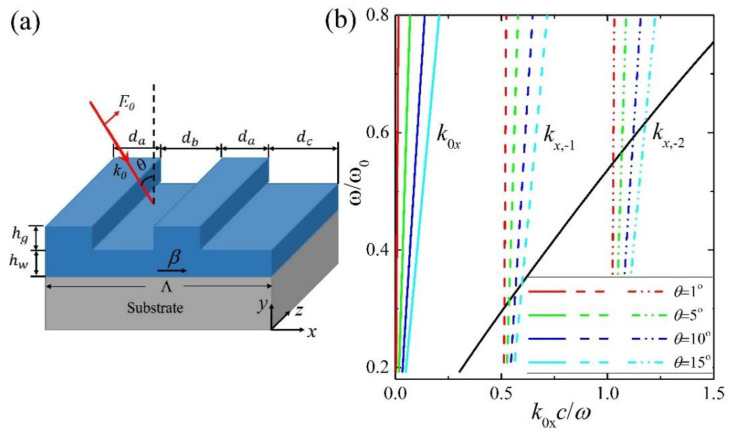
(**a**) Schematic of the GMR nanostructure of a unite cell. (**b**) The dispersion of the TE_0_ guided mode (black solid line), and *k_x_* = *k_x,i_* (*i* = −1, −2) at different incident angles, 1° (red dashed lines), 5° (green dashed lines), 10° (blue dashed lines), and 15° (cyan dashed lines), respectively.

The schematic unit cell of the SiN–GMR structure on a fused silicon substrate is shown in Figure 1a, similar to the structure reported in [26,27]. *d_a_*, *d_b_* and *d_c_* denote the width of filled and unfilled parts of grating layer, respectively. Λ represents the period of grating layer with Λ = 2 ∗ *d_a_* + *d_b_* + *d_c_*. We set *d_a_* = 0.2Λ, *d_b_* = *d* − Δ*d* and *d_c_* = *d* + Δ*d* with *d* = 0.3Λ, and *δ* = Δ*d*/*d* is an adjustable geometric parameter. The thickness of the grating layer is denoted as *h*_w_ and the waveguide layer as *h*_g_. In this paper, we set Λ = 629 nm, *h*_w_ = 320 nm, and *h*_g_ = 30 nm. The substrate has a semi-infinite thickness and is truncated, using perfect matched layer (PML) during the simulation. The parameters ensure the nanostructures of quasi-BICs at near-infrared wavelengths, which can be fetched from the commercial optical parametric oscillator (OPO) system for experimental measurements. The transverse-electric (TE) polarized light of an incident angle *θ* shines on the structure in the *oxz* plane. The wavevector of *k*_0_ is 2*π*/*λ* with *λ* as the wavelength, electric field *E*_0_, and intensity *I*_0_. 

The refractive index of SiN dependence on the local light intensity *I* is expressed as *n* = *n*_0_ + *n*_2_*I*, where *n*_0_ is the linear refractive index of SiN that was determined by the ellipsometry measurement, e.g., *n*_0_ = 1.936 at 1064 nm and 1.943 at 900 nm [28]. The value of *n*_2_ is taken from the references as 4.1 × 10^−16^ m^2^/W [29]. The local intensity *I* can be expressed as *I* = 0.5*n*ε_0_*c*|*E*_loc_|^2^, where ε_0_ is the permittivity of the vacuum, *c* is the speed of light in the vacuum, and *E*_loc_ is the local electric field in the SiN domains. The nonlinear absorption in the SiN film in the wavelength range we considered is negligible, due to the large bandgap. The numerical simulation was conducted, using FEM via the commercial software Comsol Multiphysics. The refractive index *n* of SiN under the local intensity *I* can be directly written into the SiN domains in the software. The settings are similar to those we used in Refs. [9,27].

The propagation constant *β* of the guided mode in the waveguide layer under TE polarization is determined by the following [30]:

(1) $$h_{w}\sqrt{k_{0}^{2}n_{w}^{2}-\beta^{2}}=\mathrm{atan}\left(\sqrt{\left(\beta^{2}-k_{0}^{2}n_{c}^{2}\right)/\left(k_{0}^{2}n_{w}^{2}-\beta^{2}\right)}\right)+\mathrm{atan}\left(\sqrt{\left(\beta^{2}-k_{0}^{2}n_{s}^{2}\right)/\left(k_{0}^{2}n_{w}^{2}-\beta^{2}\right)}\right)$$

where *n_w_*, *n_c_* and *n_s_* are the refractive indices of the waveguide, cladding layer and substrate, respectively. For simplicity, *n_c_* is taken as 1. The result is shown in Figure 1b, where the *ω*_0_ = 2π*c*/*h_w_* for the *y*-axis. The *x*-component of the wave vector *k_x_* is written as *k_x_* = *k*_0*x*_ = *k*_0_sin*θ* and *k_x_* = *k_x,I_* = *k*_0_sin*θ* − *iG* (*i* = ±1, ±2, …) in the air and grating layers, respectively, where *G* = 2π/Λ is the reciprocal lattice when δ ≠ 0, and becomes *G*’ = 2π/(Λ/2) as *δ* = 0. The *k*_x,i_ with different *i* under different angles of incidence is shown in Figure 1b. The crossing points satisfying *k_x_* = *k_x_*_,*i*_ = *k*_0_sin*θ* − *iG* = *β* in Figure 1b represent the phase matching condition to excite the GMR modes. These are 0.302*ω*_0_ (1059.6 nm), 0.313*ω*_0_ (1022.4 nm), 0.328*ω*_0_ (976.2 nm), and 0.344*ω*_0_ (930.8nm) at *θ* = 1°, 5°, 10°, 15°, respectively, for the negative first-order modes in the nanostructure of *δ* ≠ 0. The excitable GMR modes cannot be enabled when *δ* = 0, due to the doubled reciprocal lattice. The quasi-BICs of high *Q* factors can be realized when δ changes from zero to nonzero as discussed in Refs. [26,27].

Nonlinear TCMT is employed to analysis the reflectance spectrum of GMR consisting of Kerr media under different input intensity. An isolated optical resonator can be analyzed using TCMT as follows [31]:

(2) $$\frac{da(t)}{dt}=i(\omega_{0}-\gamma )a+<d^{*}|s_{+}>$$

(3) $$|s_{-}>=C|s_{+}>+a|d>$$

where *a*(*t*) is the amplitude of the optical resonator, *ω*_0_ is the resonant frequency, *γ* is the radiation loss rate, |*d*> is the coupling constants between each port and resonance state *a*, and *C* is the scattering matrix of the direct (non-resonant) process. |*s*_+_> and |*s*_−_> are the amplitudes of incoming and outgoing waves, respectively. In our designed GMR structure at quasi-BIC frequencies (Figure 1a), only zeroth diffraction are going out, and the other orders of diffraction are evanescent. Two-port model of TCMT are satisfied with $|s_{+}>={\left(s_{1+},s_{2+}\right)}^{T}$ and $|d>={\left(d_{1},d_{2}\right)}^{T}$, where the subscripts 1 and 2 correspond to the ports on the upper and lower half-space, respectively. The matrix of incident amplitude |*s*_+_> is written as $|s_{+}>={\left(\sqrt{I_{0}},0\right)}^{T}$ when the incident wave is excited on port 1, where *I*_0_ is the flux density of incident light. The light of time-harmonic propagation *e*^*iωt*^ is assumed, and thus, the amplitude *a*(*t*) has the form *a*(*t*) = *a e*^*iωt*^. Then, $a=d_{1}\sqrt{I_{0}}/\left[i(\omega -\omega_{0})+\gamma \right]$ is obtained from Equation (2). The outgoing amplitudes can be deduced from Equation (3) as follows [31]:

(4) $$|s_{-}>\equiv S|s_{+}>=\left[C+\frac{|d><d{|}^{*}}{i(\omega -\omega_{0})+\gamma }\right]|s_{+}>$$

where *S* is defined as the scattering matrix, and $S=C+|d><d{|}^{*}/\left[i(\omega -\omega_{0})+\gamma \right]$. The general form of *C* is expressed as follows [32]:

(5) $$C=e^{i\phi }\left(\begin{array}{cc} re^{-i\eta } & it\\ it & re^{i\eta } \end{array}\right)$$

where *r* and *t* are the absolute values of the reflection and transmission coefficients, respectively, with $r^{2}+t^{2}=1$ in the lossless media system. *φ* and *η* are real constants. The matrix *C* is linked with |*d*> through $C|d^{*}>=-|d>$ according to the energy conservation and time-reversal symmetry. The general solution of the above equation for |*d*> can be written as follows [32]:

(6) $$|d>=\left(\begin{array}{c} {}[r\alpha -i(1+t)\xi ]e^{i\frac{\phi -\eta }{2}}\\ {}[r\xi -i(1+t)\alpha ]e^{i\frac{\phi +\eta }{2}} \end{array}\right)$$

where *α* and *ξ* are two independent parameters. The relation between *α* and *ξ* can be obtained using the important equation <d|d>=2γ, which is deduced by energy conservation. That is, the following [32]:

(7) $$\alpha^{2}+\xi^{2}=\frac{2\gamma }{t^{2}+{\left(1+r\right)}^{2}}$$

From Equation (7), the intensity reflection coefficient *R* is expressed as follows:

(8) $$R=|C_{11}+\frac{d_{1}^{2}}{i(\omega -\omega_{0})+\gamma }{|}^{2}$$

where *C*_11_ is the first element in the matrix *C*, and $d_{1}^{2}$ can be directly written from Equation (6). The spectrum of reflectance *R* vs. *ω* under the angle of incidence *θ* can be obtained by numerical calculation. The *ω*_0_ and *γ* in the resonator can be calculated by eigenmode analysis. The matrix *C* related with the direct transport process can be obtained from the spectrum of reflectance *R* under the normal incidence at the BIC wavelength, and thus, the real values *r*, *t*, *φ* and *η* can be determined. By fitting the spectrum of reflection, the parameters *α* and *β* can be found. 

For the nonlinear case, i.e., the resonator is composed of Kerr media, the nonlinear TCMT equation is written as follows [32]:

(9) $$[i(\omega -\omega_{0})+\gamma ]a+i\frac{\varsigma }{2}n_{0}n_{2}\omega |a{|}^{2}a=\sqrt{I_{0}}d_{1}$$

where *n*_2_ is the nonlinear refractive index, $\varsigma ={\int_{V_{nlin}}dV|E_{x}^{BIC}|}^{4}$ with $E_{x}^{BIC}$ is the component of the electric field along the periodic direction (Figure 1a) at the BIC state, and $V_{nlin}$ is the volume of the nonlinear media. Equation (9) can be solved to obtain the amplitude of resonator *a*. Finally, the intensity reflection coefficient *R* from the structure of nonlinear media can obtained from Equation (3):

(10) $$R=|C_{11}+ad_{1}{|}^{2}$$

## 3. Results and Discussion

**Figure 2:**
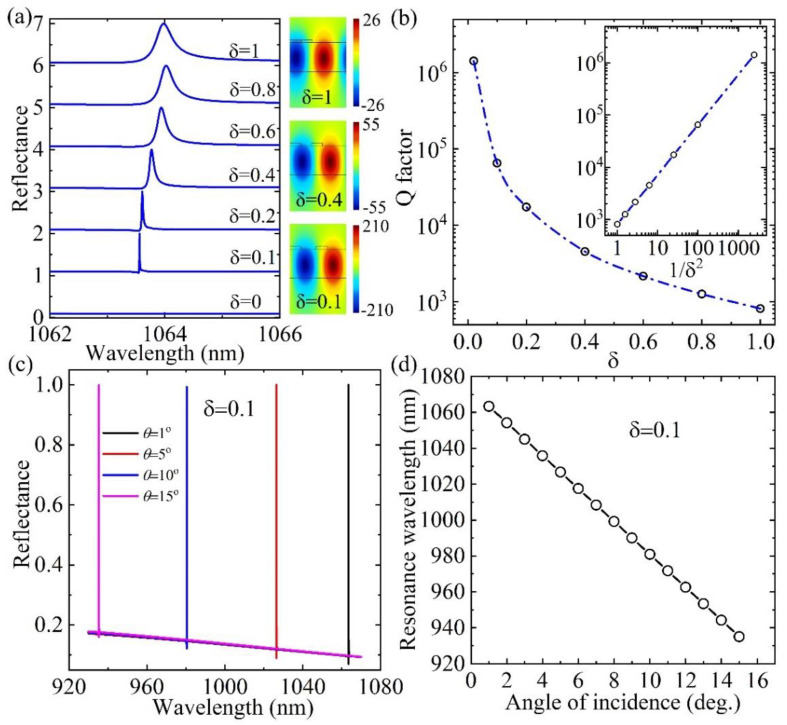
(**a**) The reflectance spectra in GMR nanostructures of different *δ* at *θ* = 1°. The electric field |*E*_y_*/E*_0_| distributions in the nanostructures of *δ* = 0.1, 0.4 and 1 at their resonance modes are shown, respectively. (**b**) *Q*-factor versus *δ*. The *Q* factor and *δ*^−2^ agree with the linear fitting well, as shown in the inset. (**c**) The reflectance in GMR nanostructure of *δ* = 0.1 versus the incident angles. (**d**) The dependence of the resonance wavelength on incident angles in the GMR nanostructure of *δ* = 0.1.

Figure 2a shows the dependence of reflectance spectra on *δ* at *θ* = 1°. The GMR wavelength λ is around 1063.56 nm at *δ* = 0.1. The resonance wavelength has a slight redshift and becomes broader when *δ* increases. It is ascribed to the change in local distributions of the refractive index in the grating layer of different *δ*. The |*E*_y_/*E*_0_| distributions in the typical nanostructures of *δ* = 0.1, 0.4 and 1 at the corresponding GMR modes are given, respectively. The maximum enhancement in the nanostructure of *δ* = 0.1 is up to 210, while the enhancement in the traditional GMR nanostructure of *δ* = 1 is only around 26. The electric field distributions at the other angles of incidence *θ* = 5°, 10°, 15° under their corresponding resonance wavelengths are similar to those at *θ* = 1°. 

The calculated *Q* factors in nanostructures of different *δ* are shown in Figure 2b. The *Q* factor increases rapidly as *δ* gradually decreases to near zero. For example, the *Q* factor is around 2.07 × 10^3^ at the traditional GMR structure of *δ* = 1 but reaches up to 6.5 × 10^4^ at *δ* = 0.1 and even 1.16 × 10^5^ at *δ* = 0.02 at the quasi-BICs. When *δ* = 0, the resonance peak vanishes completely at Δ*λ* = 0, which corresponds to the BICs. The *Q*-factor versus *δ*^−2^ has a linear relationship (inset of Figure 2b) [33]. At the same GMR structure, the resonance wavelength blueshifts with the increase in incident angles, as shown in Figure 2c for the nanostructure of *δ* = 0.1. The calculated resonance wavelength in the nanostructure of *δ* = 0.1 is at around 1026.59 nm, 980.43 nm and 935.39 nm at *θ* = 5°, 10° and 15°, respectively. The dependence of the resonance wavelength on the incident angles is summarized in Figure 2d. The resonance wavelength ranges from 1063.56 nm at 1° to 935.39 nm at 15°, which empowers the optical bistable devices to work in a broad band.

**Figure 3:**
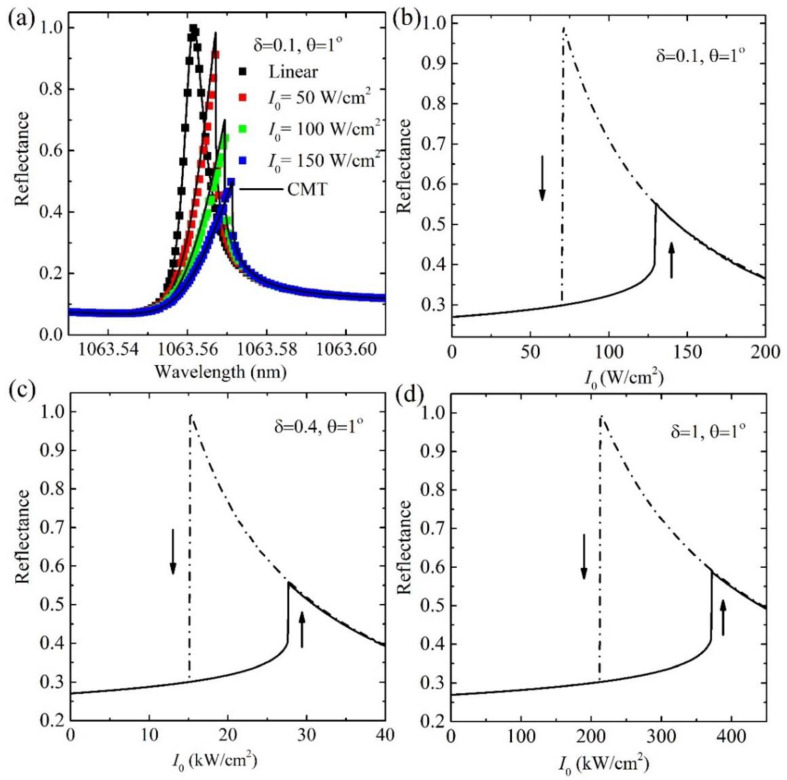
(**a**) Linear reflectance spectra under different input intensity in GMR structures of *δ* = 0.1 at *θ* = 1°. Optical bistability of linear reflectance in GMR nanostructures of different (**b**) *δ* = 0.1, (**c**) *δ* = 0.4 and (**d**) *δ* = 1 at *θ* = 1°, respectively.

When the nonlinear refraction of SiN is considered at the intense light input intensity, the reflectance is studied. Figure 3a shows the change of the reflectance spectra under different input intensities in the nanostructure of *δ* = 0.1 at *θ* = 1°. The squares are obtained from the numerical calculation using the FEM method, and the solid lines are calculated using nonlinear TCMT. The results agree well with each other. The resonance wavelength changes from 1063.56 nm under the linear dielectric condition, i.e., the dielectric nonlinearity is neglected under quite low input intensity, to 1063.57 nm at 150 W/cm^2^ when the nonlinear refraction is considered. Such a change in reflectance with the input intensity will give rise to the optical bistable response as the input intensity gradually changes from the lower to the higher value and the reverse process. 

**Figure 4:**
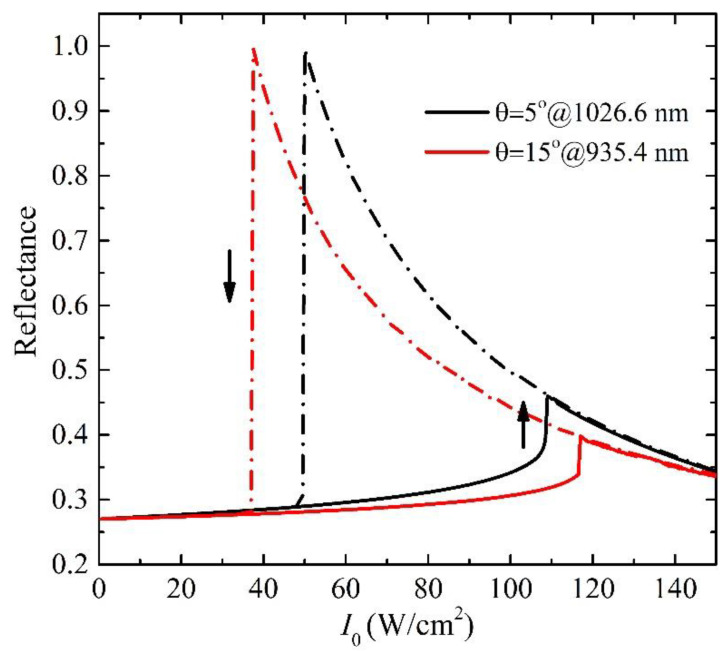
The hysteresis loops of the reflectance in the GMR nanostructures at *θ* = 5° and 15° with the working wavelength 1026.6 nm and 935.4 nm, respectively.

The evolution of reflectance in the GMR at a fixed wavelength when the input intensity gradually increases and decreases is calculated. The process is the same as used in the Refs. [8,9]. The hysteresis loops for the reflectance of two stable branches as a function of the input intensity are shown in Figure 3b–d for different structures at *θ* = 1°. The working wavelengths are chosen as the wavelengths of the linear reflectance around 27% for the different structures. The values are around 1063.57 nm and 1064.45 nm for the *δ* = 0.1 and 1, respectively. The threshold intensity *I*_th_ as the lower reflectance state jumps to the higher reflectance state is only around 130 W/cm^2^ when the *δ* = 0.1. The *I*_th_ increases with the increase in *δ* due to the reduced local field enhancement. At *δ* = 1 for the traditional GMR structure, the *I*_th_ is around 380 kW/cm^2^, which is three orders of magnitude larger than that in the nanostructure of *δ* = 0.1 at the quasi-BIC state. The ultra-low threshold intensity at the level of ~100 W/cm^2^ at the nanostructure of *δ* = 0.1 is three to six orders of magnitude lower than that reported in the other typical GMR structures [21,22,23,34], metallic or graphene surface plasmon resonance structures [8,9,10,11] as well as the photonic crystals [6,7]. Further, the working wavelength is tunable by the angle of incidence at the same structure, while the threshold intensity is almost unchanged at the same starting reflectance. For example, the working wavelength can be tuned to be 1026.6 nm, 980.44 nm, and 935.4 nm at the structure of *δ* = 0.1 of reflectance 27% when *θ* = 5°, 10°, and 15°, respectively, and optical bistable loops are similar to those at *θ* = 1° with the ultra-low threshold intensity around 100 W/cm^2^. Figure 4 shows the hysteresis loops of the reflectance at the angle of incidence *θ* = 5° and 15°, respectively.

**Figure 5:**
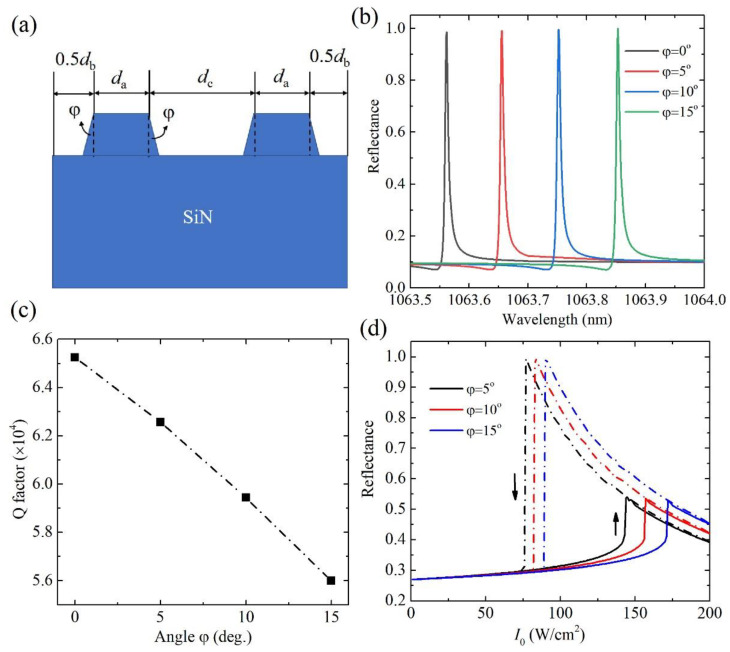
(**a**) Schematic of a unit cell of the GMR nanostructure of round corners of grating. The radius of round corner is noted as r. (**b**) Reflectance spectra, (**c**) *Q*-factor and (**d**) optical bistability in GMR structures (*δ* = 0.1) of different radius of round corners at *θ* = 1°.

We finally analyze the effect of typical defects of nanostructure, which may be introduced during the nanofabrication, on the linear optical properties and optical bistable behaviors. The simulations are also performed, using the FEM solver. The settings are the same as those employed for the ideal structures, just changing the idea geometry into the corresponding defects. We first consider that the corners of the grating are slightly rounded to radius r during the fabrication, as shown in Figure 5a. The linear reflectance spectra of different r at *θ* = 1° are shown in Figure 5b. The resonance wavelength has a clear blueshift with the increase in r. The increase in r leads to the reduction in an effective refractive index in the grating-air layer or the cladding layer, and thus increases the propagation constant β of the waveguide layer according to Equation (1). So, the blueshift resonance wavelength happens. Although the radius of the round corner is up to 20 nm, the shift of resonance wavelength is only about 0.15 nm. The *Q*-factor has a slight increase with the increase in r as shown in Figure 5c. The optical bistability of reflectance in the GMR nanostructure of round corner r = 5, 10, and 20 nm is shown in Figure 5d, respectively. The working wavelengths are all set at the reflectance of around 27%. The hysteresis loops for the reflectance of reduced intensity thresholds due to the slight increased *Q*-factor are observed. The intensity threshold is around 110 W/cm^2^ when r = 20 nm, and the change in the intensity threshold is within 20%, compared with that in ideal structures. Those results indicate that the round corner of the grating in the designed GMR during the nanofabrication will not affect the linear optical properties and optical bistable response too much.

**Figure 6:**
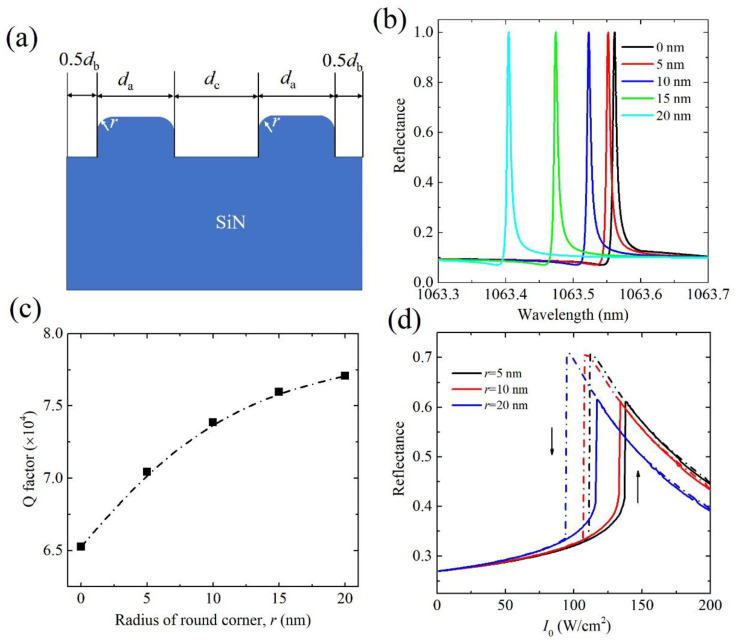
(**a**) Schematic of a unit cell of the GMR nanostructure when the rectangular cross section of grating is deviated. (**b**) Reflectance spectra, (**c**) *Q*-factor and (**d**) optical bistability in GMR structures (*δ* = 0.1) of different radii of round corners at *θ* = 1°.

We next discuss the effect of a small deviation of rectangular cross section of grating, as shown in Figure 6a, on the linear reflectance and optical bistable response. The upper side of grating strip is kept as *d*_a_ while the bottom side becomes larger with the tilted angle *φ*. The linear reflectance spectra in the nanostructures of different *φ* are shown in Figure 6b. The redshift of the resonance wavelength is observed with the increase in tilted angle *φ*. This can also be explained from Equation (1), i.e., the increase in the effective refractive index of the cladding layer of the waveguide will reduce the propagation constant *β* of the waveguide layer. The resonance wavelength has a shift around 0.3 nm when the tilted angle *φ* is up to 15°. The *Q*-factor slightly decreases with the increase in *φ* as shown in Figure 6c. The hysteresis loops of the reflectance in the GMR nanostructure of different tilted angle *φ* are shown in Figure 6d. The intensity threshold has a slight increase with the increase in *φ* due to the decrease in the *Q*-factor. The intensity threshold arrives at around 170 W/cm^2^ at *φ* = 15°, which is still at the level of 100 W/cm^2^. So, the main conclusions on linear optical properties and optical bistable response in the designed GMR of the deviated rectangular cross section of grating are kept. The results of structures of the other defects, such as the bottom side of the grating, which is kept as *d*_a_ while the upper side is reduced, and the deviated rectangular cross section of the grating of the round corners, are not fully presented here.

**Figure 7:**
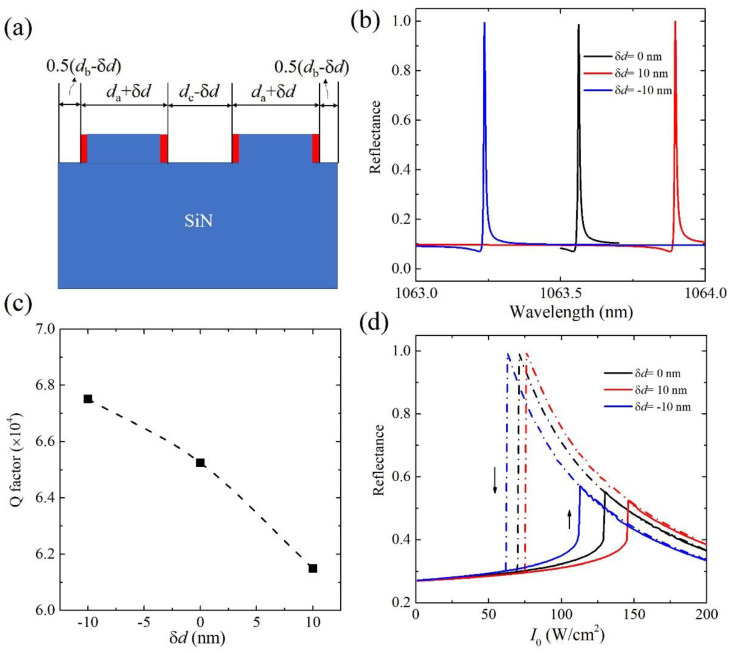
(**a**) Schematic of a unit cell of the GMR nanostructure if the designed width *d*_a_ has an error *δd* introduced during the nanofabrication. The red parts in the grating represent the error *δd*. (**b**) Reflectance spectra, (**c**) *Q*-factor and (**d**) optical bistability in GMR nanostructures (*δ* = 0.1) of *δd* = ± 10 nm at *θ* = 1°.

The influence of the deviation of the designed width of grating *d*_a_ during the impractical nanofabrication (as shown in Figure 7a) on the linear and bistable response is finally discussed. The typical error *δd* of ±10 nm is considered. The linear reflectance and *Q*-factor in the deviated nanostructure are shown in Figure 7b,c, respectively. Compared with the nanostructure of the designed width *d*_a_, the deviation *δd* = 10 nm and −10 nm will lead the resonance wavelength to redshift and blueshift, respectively. Such shifts can also be expected considering Equation (1), i.e., the increased (reduced) width of the grating will increase (decrease) the effective refractive index of cladding layer of waveguide, and then reduce (increase) the propagation constant *β* of waveguide layer to produce the reshifted (blueshifted) resonance mode. The *Q*-factor has a slight increase when *δd* = −10 nm and decrease when *δd* = 10 nm. The reflectance hysteresis loops in the GMR nanostructure of *δd* = ± 10 nm are shown in Figure 7d. For comparison, the hysteresis loop from the GMR of designed *d*_a_ is also presented. The intensity threshold has a slight increase in the nanostructure of *δd* = 10 nm, which decreases when *δd* = −10 nm due to the change in the *Q*-factor. The intensity threshold is still at the level of 100 W/cm^2^, indicating that the performance of the optical bistable device is also kept under the reasonable deviation of the designed width of the grating.

## 4. Conclusions

In summary, we investigate the optical bistability in GMR nanostructures of quasi-BICs states. The broadband and ultra-low intensity of bistability in the feasible structures of quasi-BICs are obtained. The large-scale nanostructures can be fabricated using state-of-the-art nanofabrication techniques, such as electron-beam lithography, nano-imprint lithography and the etching method, and satisfy the high-throughput and cost-efficient requirements. The tolerance of defects in the nanostructures makes the experimental measurements and optical devices more practical. Besides SiN being used as the Kerr medium in the article, the other dielectric materials of larger nonlinear refraction, such as AlGaAs and SiC, can further reduce the intensity threshold of optical bistability for high-performance all-optical devices.

## Data Availability

All data are contained within the article.
